# Correction: Formononetin in Radix Hedysari extract-mediated green synthesis of gold nanoparticles for colorimetric detection of ferrous ions in tap water

**DOI:** 10.1039/d1ra90152d

**Published:** 2021-10-08

**Authors:** Xinyue Chen, Jiahui Ji, Gengen Shi, Zhiyuan Xue, Xianglin Zhou, Lianggong Zhao, Shilan Feng

**Affiliations:** Institute of Pharmaceutical Analysis, School of Pharmacy, Lanzhou University Lanzhou 730000 P. R. China fengshl@lzu.edu.cn chenxinyue888@126.com 13636442199@163.com shigg18@lzu.edu.cn 13679449239@163.com 1195533044@qq.com +86 931 8915686; The Second Hospital of Lanzhou University Lanzhou 730030 P. R. China lzxtj2008@hotmail.com +86 931 8942460

## Abstract

Correction for ‘Formononetin in Radix Hedysari extract-mediated green synthesis of gold nanoparticles for colorimetric detection of ferrous ions in tap water’ by Xinyue Chen *et al.*, *RSC Adv.*, 2020, **10**, 32897–32905, DOI: 10.1039/D0RA05660J.

The authors regret that the funding information for the original article was incorrectly given. The corrected funding information is as follows.

The authors greatly appreciate financial support from the National Natural Science Foundation of China (No. 81703664) and the China Postdoctoral Science Foundation (No. 2019M663855).

The Royal Society of Chemistry apologises for these errors and any consequent inconvenience to authors and readers.

## Supplementary Material

